# Comparative Materials-Level Evaluation of 3′- and 5′-Thiol DNA Aptamer Conjugation on Gold Nanospheres and Nanoflowers: Apparent DNA Loading Output, Morphology Retention, and Qualitative Salt-Challenge Response

**DOI:** 10.3390/s26103076

**Published:** 2026-05-13

**Authors:** Jingchun Sun, Linbing Zhang, David Gonçalves, Shaoping Kuang, Hongsheng Yang

**Affiliations:** 1 College of Environment and Safety Engineering, Qingdao University of Science and Technology, Qingdao 266042, China; sunjingchun@qdio.ac.cn; 2 Laboratory of Marine Ecology and Environmental Sciences, Institute of Oceanology, Chinese Academy of Sciences, Qingdao 266000, China; 3 Laboratory for Marine Ecology and Environmental Science, Qingdao Marine Science and Technology Center, Qingdao 266237, China; 4 State Key Laboratory of Breeding Biotechnology and Sustainable Aquaculture, Institute of Oceanology, Chinese Academy of Sciences, Qingdao 266000, China; 5 Institute of Science and Environment, University of Saint Joseph, Macau, China; 6 William James Center for Research, ISPA—Instituto Universitário, 1149-041 Lisbon, Portugal

**Keywords:** gold nanoparticles, gold nanoflowers, thiolated DNA aptamer, aptamer conjugation, salt-aging, apparent DNA loading, zeta potential, qualitative salt tolerance

## Abstract

**Highlights:**

**What are the main findings?**

- Under matched preparation conditions, AuNP samples showed higher apparent aptamer conjugation output than AuNF samples, with modest differences between 3′- and 5′-thiol constructs.
- Aptamer modification increased hydrodynamic diameter, shifted zeta potential toward more negative values, and preserved particle morphology after conjugation.

**What are the implications of the main findings?**

- Aptamer-functionalized AuNPs and AuNFs showed improved qualitative visual tolerance to salt-induced aggregation relative to bare particles under the tested conditions.
- The study provides preliminary materials-level information for evaluating thiolated aptamer conjugation behavior and may support future glyphosate aptasensor optimization.

**Abstract:**

Gold nanospheres (AuNPs) and gold nanoflowers (AuNFs) are widely used as platforms for DNA aptamer functionalization, while conjugation behavior and colloidal tolerance remain important factors affecting subsequent sensing-oriented optimization. In this study, 82-nt thiolated DNA aptamer constructs bearing either 3′-SH or 5′-SH terminal modification were immobilized onto citrate-stabilized AuNPs and AuNFs under matched stepwise salt-aging conditions. Apparent nanoparticle-associated DNA output was estimated by Qubit-based measurement of unbound ssDNA in the supernatant and expressed as mass-based loading output (ng). Under the tested stock-dispersion conditions, AuNP samples showed higher apparent conjugation output than AuNF samples. Specifically, the apparent conjugation yields for AuNPs were 80.65 ± 1.64% (3′-SH) and 84.76 ± 1.98% (5′-SH), whereas those for AuNFs were 66.64 ± 3.36% (3′-SH) and 73.65 ± 1.36% (5′-SH). The corresponding apparent DNA loading outputs were 2329.7 ± 47.4 ng and 2448.7 ± 57.1 ng for AuNPs, and 1925.1 ± 97.0 ng and 2127.4 ± 39.3 ng for AuNFs. DLS size increases and zeta potential shifts toward more negative values were consistent with the formation of a DNA-associated interfacial layer, while TEM images supported morphology retention after conjugation. A qualitative visual salt-challenge assessment indicated that aptamer-functionalized nanoparticles displayed improved resistance to salt-induced aggregation relative to bare particles under the tested conditions. Because the commercially supplied AuNP and AuNF dispersions were not normalized to identical particle number or accessible surface area, the reported values should be interpreted as comparative apparent outputs rather than intrinsic loading capacities. Within this scope, the present study provides a convenient preliminary materials-level evaluation of thiolated aptamer conjugation behavior and may support future glyphosate aptasensor optimization.

## 1. Introduction

Gold nanoparticles (AuNPs) are widely used as nanoscale building blocks in materials science because of their localized surface plasmon resonance, chemical stability, and well-established surface functionalization chemistry [1,2,3]. Beyond composition alone, the morphology of gold nanostructures—ranging from smooth nanospheres to branched nanoflowers—provides an additional means of tuning interfacial area, local curvature, and surface heterogeneity, all of which may influence their behavior in optical, bioanalytical, and nano–bio interface applications [2,4,5,6]. Consequently, morphology-controlled gold nanostructures remain of sustained interest not only for signal generation, but also for understanding how structural features affect interfacial modification and downstream analytical integration [4,5,6]. In parallel, nucleic acid aptamers provide programmable molecular recognition, predictable structure–function behavior, and facile chemical modification, making them attractive ligands for gold nanostructure functionalization [7,8,9]. Thiolated DNA aptamers can be immobilized on gold surfaces through Au–S interactions, enabling the construction of hybrid nanointerfaces in which ligand presentation, colloidal behavior, and surface-associated charge collectively affect subsequent analytical performance [10,11,12]. In recent years, aptamer-functionalized nanomaterials have also been increasingly integrated into point-of-care and on-site sensing formats, including miniaturized and automated microfluidic platforms that improve sample handling, reaction control, and rapid detection capability [8]. For example, aptamer–AuNP colorimetric assays have already been combined with centrifugal microfluidic systems to enable automated and portable detection workflows, highlighting the importance of robust upstream conjugation chemistry for reliable downstream integration [13]. Nevertheless, in many such systems, practical performance remains constrained not only by aptamer recognition itself, but also by conjugate stability, interfacial reproducibility, and tolerance to changing ionic environments [14,15,16,17,18]. A key step in preparing DNA–gold conjugates is the immobilization of thiolated oligonucleotides onto citrate-stabilized gold surfaces. The widely used salt-aging strategy gradually increases ionic strength to screen electrostatic repulsion among DNA strands and thereby promote interfacial loading through Au–S anchoring [19,20,21,22]. However, the outcome of this process is sensitive to particle morphology, surface chemistry, aggregation tendency, and purification conditions, particularly for larger or anisotropic nanostructures [20,21,22,23]. From a practical characterization perspective, nanoparticle-associated DNA is often evaluated through combined but largely indirect indicators such as hydrodynamic size, zeta potential, and electron microscopy, whereas direct comparison of loading behavior across different nanostructure morphologies remains challenging [23,24,25,26,27,28]. In particular, there is still a lack of studies that compare, under matched preparation conditions, how terminal thiol placement (3′ vs. 5′) and gold nanostructure morphology (spherical vs. flower-like) jointly influence apparent conjugation behavior using the same aptamer sequence and the same workflow. At the same time, rigorous particle-number- or surface-area-normalized loading comparisons remain an important unresolved issue in the field [29,30,31,32,33]. In this work, thiolated DNA aptamer constructs bearing either 3′-SH or 5′-SH terminal modification were conjugated onto commercially supplied gold nanospheres and gold nanoflowers using a matched stepwise salt-aging workflow. The resulting systems were comparatively evaluated in terms of apparent Qubit-based DNA association output, hydrodynamic size, zeta potential, morphology retention, and qualitative visual response to salt challenge. Rather than serving as a complete validation of biosensor performance, this study was designed as a convenient preliminary materials-level evaluation of thiolated aptamer conjugation behavior on gold nanostructures. Within this scope, the work aims to clarify how terminal thiol placement and nanostructure morphology influence apparent conjugation-related outcomes under standardized preparation conditions, and to provide an initial basis for future optimization of glyphosate aptamer-functionalized sensing materials.

## 2. Materials and Methods

### 2.1. Materials and Reagents

Gold nanospheres (AuNPs) and gold nanoflowers (AuNFs) were purchased from Nanjing Shennoqing Biotechnology Co., Ltd. (Nanjing, China) (AuNP: SNQ-202409-AuNP; AuNF: SNQ-202409-AuNF). A previously reported glyphosate-binding DNA aptamer sequence (82 nt; Qin et al. [28]) was synthesized with terminal thiol modification at either the 3′ end or the 5′ end to generate two thiolated aptamer constructs (3′-SH and 5′-SH) for comparative conjugation evaluation. The aptamer sequence (5′→3′) was as follows: 5′-GCTAGACGATATTCGTCCATCCGAGCCCGTGGCGGGCTTTAGGACTCTGCGGGCTTCGCGGCGCTGTCAGACTGAATATGTC-3′.

Tris(2-carboxyethyl)phosphine hydrochloride (TCEP) was used to reduce disulfide-protected oligonucleotides and generate free thiol groups before conjugation. Sodium chloride (NaCl) was used for stepwise salt-aging to gradually increase ionic strength during surface immobilization. Sodium dodecyl sulfate (SDS) was included as a stabilizing surfactant during conjugation and purification. Unless otherwise specified, all reagents were used as received, and nuclease-free water was used throughout the experiments.

### 2.2. Instruments

The major instruments used in this study are listed in Table 1. Qubit 4.0 (Thermo Fisher Scientific, Waltham, MA, USA) was used for ssDNA quantification in the supernatant, Nano ZS (Malvern Panalytical, Malvern, Worcestershire, UK) was used for dynamic light scattering (DLS) and zeta potential measurements, and JEM-2100 transmission electron microscopy (JEOL Ltd., Akishima, Tokyo, Japan) was used for morphology observation. Unless otherwise specified, the instruments were operated according to the manufacturers’ recommended procedures.

### 2.3. Stepwise Salt-Aging Conjugation of Thiolated Aptamers

Before conjugation, thiolated aptamer constructs were activated by reduction with TCEP to generate free terminal thiol groups for Au–S surface immobilization. Briefly, 8.3 μL of 20 mM TCEP was added to the dried DNA sample and the mixture was diluted to 0.1 mL with nuclease-free water (DNA concentration approximately 10 OD/mL). The solution was incubated at room temperature for 1 h.

For conjugation, 1 mL of AuNP or AuNF dispersion was gently mixed with 10 μL of the activated aptamer solution and 5 μL of 4% (*w*/*v*) SDS. The mixture was incubated at room temperature overnight (approximately 16 h) to allow adsorption and thiol-mediated surface association with the gold nanostructures.

Stepwise salt-aging was then performed to gradually increase ionic strength and reduce electrostatic repulsion among surface-associated DNA strands. A 2 M NaCl solution was sequentially added in four steps: 25 μL (~0.05 M), 27 μL (~0.10 M), 58 μL (~0.20 M), and 65 μL (~0.30 M). After each addition, the samples were gently mixed and equilibrated at room temperature for 8 h. During this process, the dispersion was visually checked for obvious aggregation. When transient color instability was observed, brief bath sonication (10 s) was applied to facilitate redispersion before continuing the procedure. SDS was retained in the system as a stabilizing surfactant during increasing ionic strength.

After salt-aging, the conjugates were purified by centrifugation at 13,000 rpm for 20 min, followed by removal of the supernatant and resuspension of the pellet in 0.01% SDS solution. This centrifugation/resuspension process was repeated twice, and the final pellet was resuspended in 1 mL of ultrapure water for subsequent characterization. The purification procedure was applied identically to all groups to improve inter-sample comparability. However, the purpose of this step was to reduce free or loosely associated DNA in a standardized manner, rather than to claim absolute separation between irreversibly surface-bound DNA and all weakly associated DNA species.

### 2.4. Qubit-Based Estimation of Apparent Conjugation Output

The concentration of residual unbound ssDNA in the collected supernatant was measured using a Qubit 4.0 fluorometer with the Qubit ssDNA assay kit. Measurements were performed in quadruplicate, and values are reported as mean ± SD (*n* = 4). Based on a mass-balance approach, the amount of nanoparticle-associated DNA remaining after standardized purification was estimated by subtracting the measured supernatant ssDNA from the total DNA input.

The apparent nanoparticle-associated DNA amount was calculated as:

Apparent nanoparticle-associated DNA (ng) = total DNA input (ng) − supernatant ssDNA (ng)

The apparent conjugation yield was calculated as:

Apparent conjugation yield (%) = [apparent nanoparticle-associated DNA (ng)/total DNA input (ng)] × 100

Because this calculation is based on residual ssDNA measured after standardized centrifugation and washing, the resulting values should be interpreted as apparent mass-based conjugation outputs, rather than absolute measurements of irreversibly chemisorbed DNA alone.

### 2.5. Particle Characterization

#### 2.5.1. Dynamic Light Scattering (DLS) and Zeta Potential

Hydrodynamic diameter and zeta potential of bare and aptamer-modified nanoparticles were measured using the Nano ZS system. Samples were diluted 1:10 in nuclease-free water and analyzed at room temperature. Measurements were performed in quadruplicate (*n* = 4), and data are reported as mean ± standard deviation. These measurements were used as practical interfacial characterization indicators for comparative evaluation of particle-associated DNA modification.

#### 2.5.2. Transmission Electron Microscopy (TEM)

For morphology observation, 10 μL of each sample was drop-cast onto carbon-coated copper grids and dried under ambient conditions for 10–15 min. TEM imaging was used to assess particle morphology before and after conjugation and to examine whether obvious deformation, fragmentation, or large-scale aggregation occurred during the preparation process.

#### 2.5.3. Qualitative Visual Salt-Challenge Assessment

Bare and aptamer-functionalized nanoparticles were exposed to NaCl solution at final concentrations of 0.1–0.3 M and monitored over 7 days at predefined time points (Days 0, 1, 3, 5, and 7). Changes in sample appearance, including visible color change and macroscopic aggregation behavior, were recorded photographically. This experiment was intended as a qualitative visual comparison of salt-challenge response under the tested conditions, rather than a quantitative kinetic stability analysis.

### 2.6. Statistical Analysis

Data are presented as mean ± standard deviation (SD). Statistical analysis was performed using GraphPad Prism version 8.0. For comparisons among groups, one-way analysis of variance (ANOVA) followed by Tukey’s multiple-comparison test was used where appropriate. Where the current dataset was intended for comparative description rather than formal inference, the results are described conservatively to avoid overinterpretation.

## 3. Results

### 3.1. Qubit-Based Apparent DNA Association Output Under Matched Preparation Conditions

Residual unbound single-stranded DNA (ssDNA) in the collected supernatant was quantified using the Qubit ssDNA assay, and the nanoparticle-associated DNA fraction remaining after standardized purification was estimated by mass balance. Values are reported as mean ± SD (*n* = 4). Because the AuNP and AuNF stock dispersions were used as supplied and were not normalized to identical particle number or total accessible surface area, the resulting values are presented here as apparent mass-based conjugation outputs under matched preparation conditions.

Under the tested conditions, AuNP samples showed higher apparent nanoparticle-associated DNA output than AuNF samples (Table 2). For AuNPs, the apparent DNA-associated mass was 2329.7 ± 47.4 ng for the 3′-SH construct and 2448.7 ± 57.1 ng for the 5′-SH construct. For AuNFs, the corresponding values were 1925.1 ± 97.0 ng for the 3′-SH construct and 2127.4 ± 39.3 ng for the 5′-SH construct. The corresponding apparent conjugation yields were 80.65 ± 1.64% and 84.76 ± 1.98% for AuNP–3′SH and AuNP–5′SH, respectively, and 66.64 ± 3.36% and 73.65 ± 1.36% for AuNF–3′SH and AuNF–5′SH, respectively.

Within each particle type, the 5′-SH construct showed slightly higher apparent output than the 3′-SH construct under the present workflow. However, these data should be interpreted as comparative outputs obtained after standardized washing and purification, rather than as absolute measurements of irreversibly surface-bound DNA only.

### 3.2. Hydrodynamic Size and Zeta Potential Changes After Conjugation

**Figure 1:**
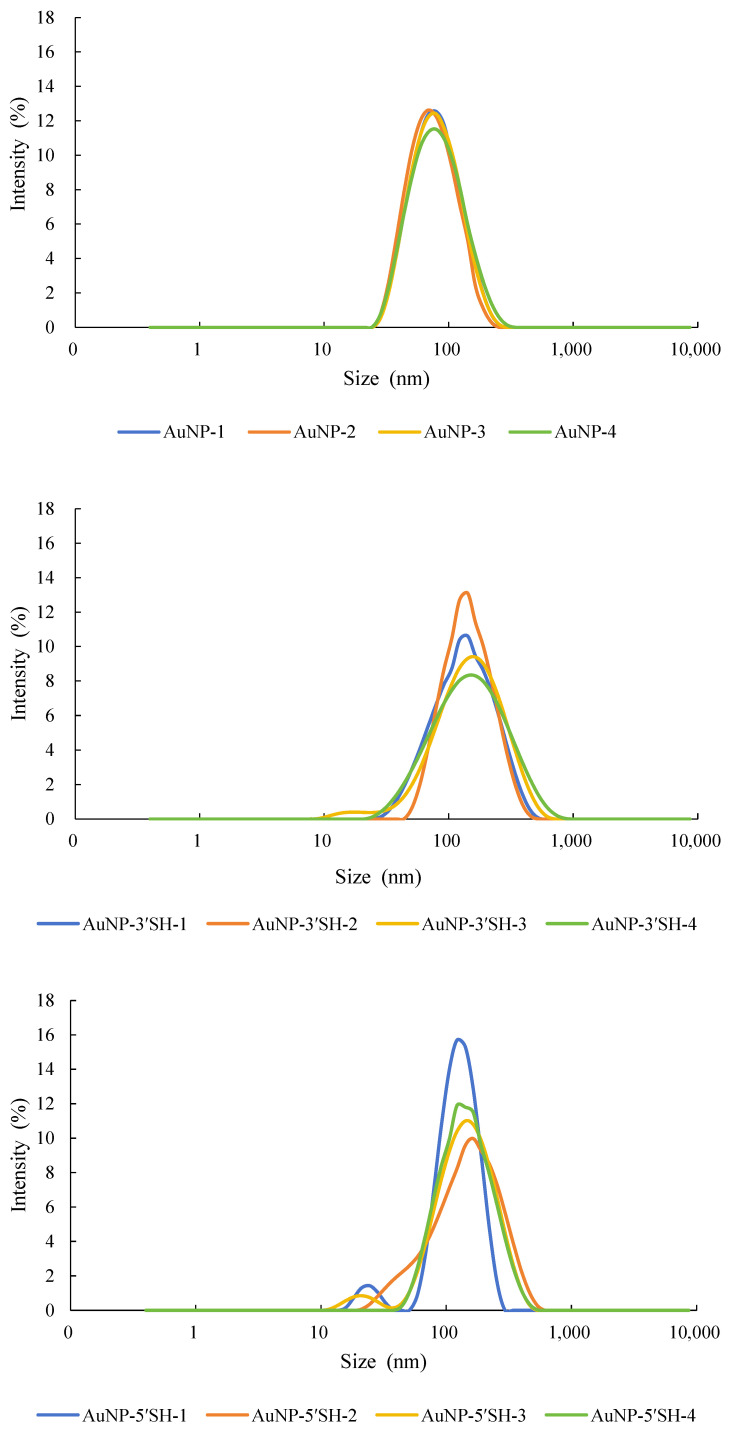
Hydrodynamic diameter of bare and aptamer-modified AuNPs measured by DLS. DLS analysis showed an increase in hydrodynamic size after modification, consistent with formation of a hydrated DNA-associated interfacial layer.

**Figure 2:**
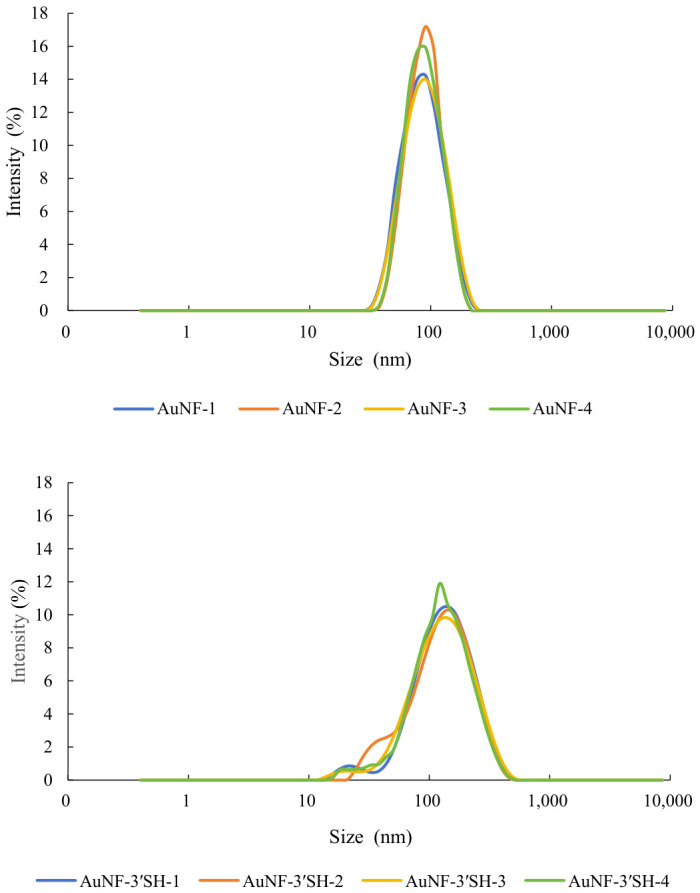

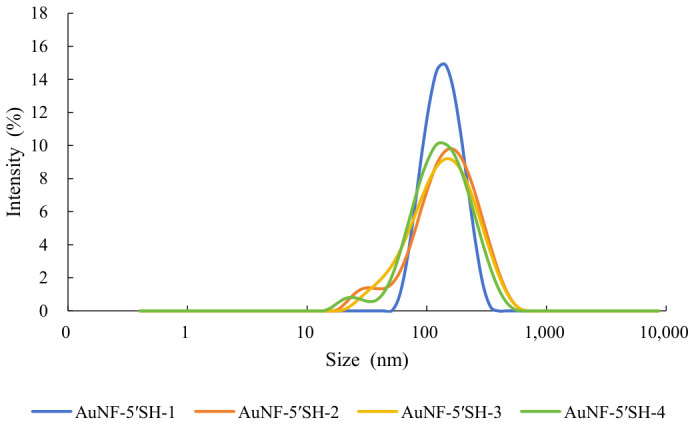
Hydrodynamic diameter of bare and aptamer-modified AuNFs measured by DLS. DLS analysis showed an increase in hydrodynamic size after modification, consistent with formation of a hydrated DNA-associated interfacial layer.

Dynamic light scattering (DLS) measurements showed a clear increase in hydrodynamic diameter after aptamer modification for both AuNPs and AuNFs (Table 3). For AuNPs, the hydrodynamic diameter increased from 70.41 ± 1.10 nm for bare particles to 118.30 ± 2.04 nm for the 3′-SH construct and 119.10 ± 1.06 nm for the 5′-SH construct (Figure 1). For AuNFs, the diameter increased from 70.35 ± 1.27 nm for bare particles to 108.80 ± 2.67 nm for the 3′-SH construct and 115.30 ± 3.18 nm for the 5′-SH construct (Figure 2). These changes are consistent with the formation of a hydrated interfacial layer after aptamer association.

**Figure 3:**
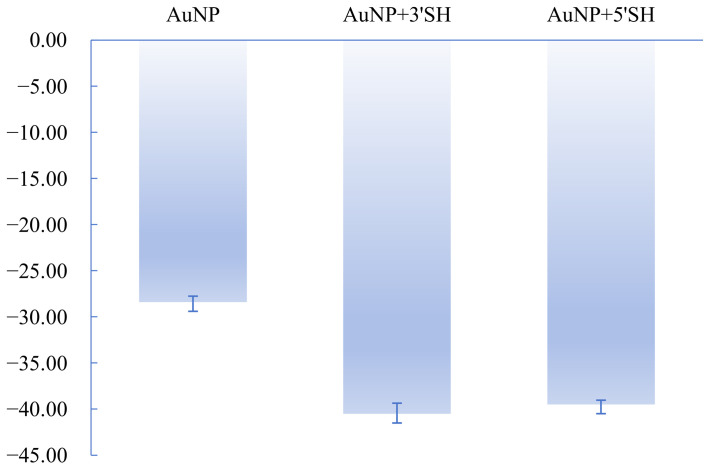
Zeta potential of bare and aptamer-modified AuNPs. The zeta potential shifted toward more negative values after modification, consistent with the contribution of surface-associated DNA.

**Figure 4:**
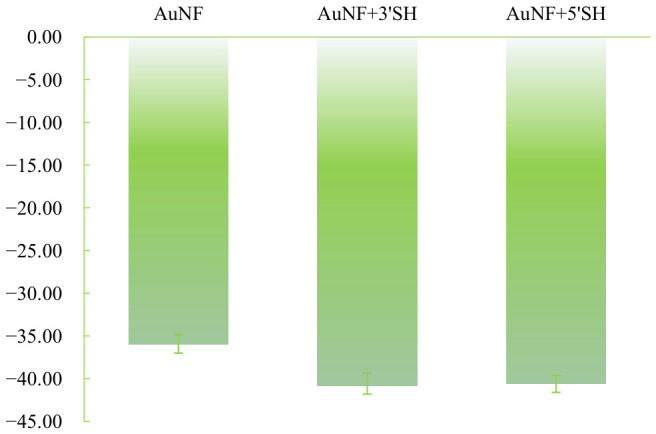
Zeta potential of bare and aptamer-modified AuNFs (mean ± SD, *n* = 4). The zeta potential shifted toward more negative values after modification, consistent with the contribution of surface-associated DNA.

Zeta potential measurements also shifted toward more negative values after aptamer modification. For AuNPs, the zeta potential changed from −28.40 ± 0.64 mV for bare particles to −40.80 ± 0.59 mV for the 3′-SH construct and −41.50 ± 0.66 mV for the 5′-SH construct (Figure 3). For AuNFs, the corresponding values changed from −36.00 ± 0.45 mV for bare particles to −40.55 ± 0.66 mV for the 3′-SH construct and −39.98 ± 0.89 mV for the 5′-SH construct (Figure 4). The observed shift toward more negative zeta potential is consistent with increased contribution from surface-associated DNA.

Although the apparent DNA association outputs differed among groups, the final zeta potential values for aptamer-modified AuNPs and AuNFs converged to a relatively narrow range near −40 mV. This result indicates that zeta potential can serve as a useful comparative indicator of interfacial modification status, while further interpretation of absolute ligand coverage requires caution.

### 3.3. TEM Imaging and Morphology Retention

**Figure 5:**
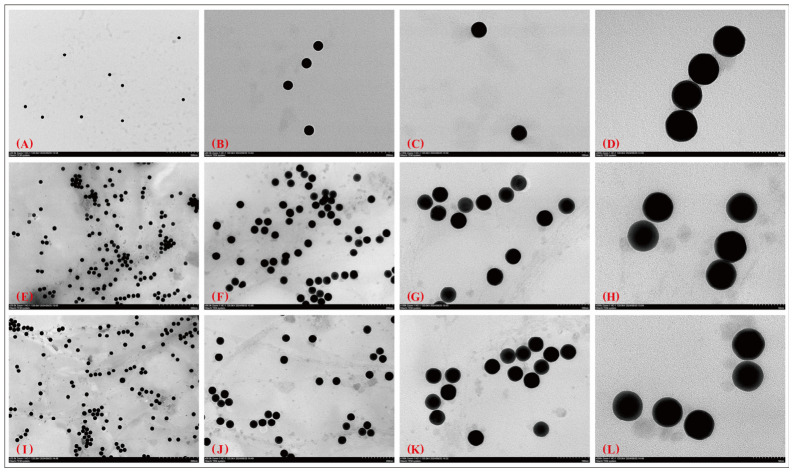
TEM images of bare and aptamer-modified AuNPs. (**A**–**D**) Bare AuNPs at different magnifications; (**E**–**H**) AuNPs modified with the 3′-SH construct; (**I**–**L**) AuNPs modified with the 5′-SH construct. Representative images at different magnifications are shown to compare particle morphology before and after modification. The spherical morphology of AuNPs was retained after aptamer association.

**Figure 6:**
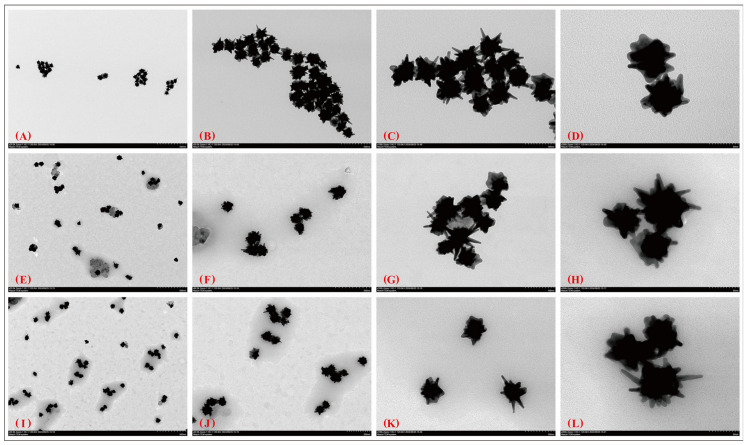
TEM images of bare and aptamer-modified AuNFs. (**A**–**D**) Bare AuNFs at different magnifications; (**E**–**H**) AuNFs modified with the 3′-SH construct; (**I**–**L**) AuNFs modified with the 5′-SH construct. Representative images at different magnifications are shown to compare particle morphology before and after modification. The branched morphology of AuNFs was retained after aptamer association, and no obvious large-scale structural disruption was observed in the representative images.

Transmission electron microscopy (TEM) images showed that the aptamer-modified nanoparticles retained their overall characteristic morphologies after conjugation. AuNPs remained predominantly spherical, with an inorganic core size of approximately 50 nm, whereas AuNFs retained their branched morphology with an effective diameter of approximately 70–100 nm (Figure 5 and Figure 6). No obvious deformation, fragmentation, or large-scale aggregation was observed in the representative images after aptamer modification.

### 3.4. Qualitative Visual Salt-Challenge Response

A qualitative visual salt-challenge assessment was performed to compare the macroscopic colloidal response of bare and aptamer-modified AuNPs and AuNFs under elevated ionic strength. Samples were exposed to NaCl solution at a final concentration of 0.2 M and monitored over 7 days.

**Figure 7:**
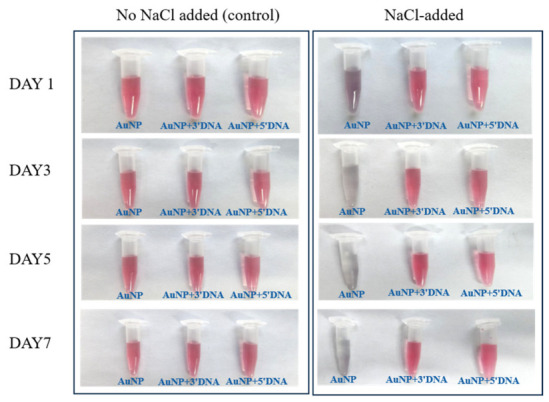
Qualitative visual salt-challenge response of bare and aptamer-modified AuNPs under 0.2 M NaCl. Photographs showing the visual appearance of bare and DNA-modified AuNPs during 7 days of NaCl exposure. Bare AuNPs showed progressive visible aggregation-associated color change, whereas modified samples showed no obvious visual color change under the tested conditions.

For AuNPs, no obvious visible color change was observed in the groups without added NaCl over the 7-day period, indicating that the dispersions remained visually stable under low-salt conditions. In contrast, the bare AuNPs exposed to NaCl showed a distinct color change from red to purple on Day 1, followed by progressive fading until the suspensions became nearly transparent, consistent with salt-induced aggregation (Figure 7). By comparison, the 3′-DNA- and 5′-DNA-modified AuNPs showed no obvious visible color change over the same period.

**Figure 8:**
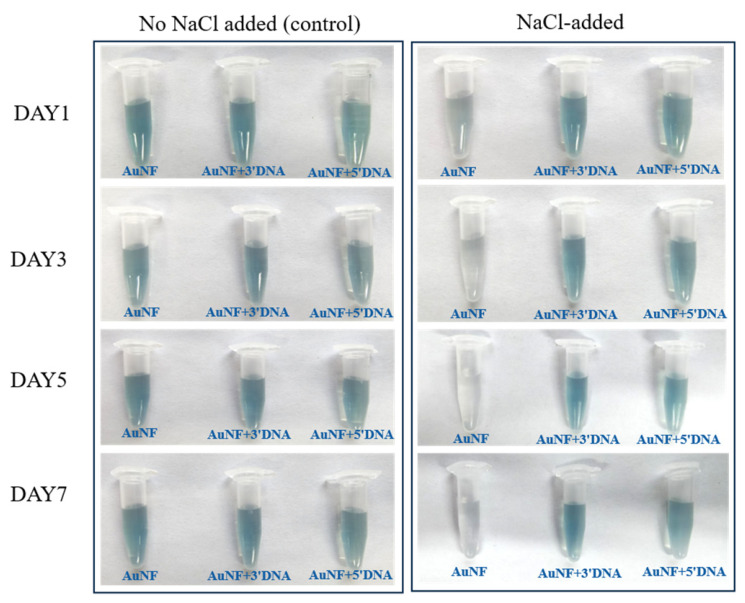
Qualitative visual salt-challenge response of bare and aptamer-modified AuNFs under 0.2 M NaCl. Photographs showing the visual appearance of bare and DNA-modified AuNFs during 7 days of NaCl exposure. Bare AuNFs showed progressive visible aggregation-associated color change, whereas modified samples showed no obvious visual color change under the tested conditions.

A similar qualitative pattern was observed for AuNFs. The groups without added NaCl remained visually stable over 7 days, whereas the bare AuNFs exposed to NaCl changed from blue to gray on Day 1 and gradually became nearly transparent with time (Figure 8). In contrast, the 3′-DNA- and 5′-DNA-modified AuNFs did not show obvious visible color change under the same conditions.

Taken together, these observations indicate that aptamer modification was associated with improved qualitative visual tolerance to salt-induced aggregation under the tested conditions. Because this assessment was based on photographic observation rather than time-resolved DLS or UV–Vis measurements, the results should be interpreted as qualitative comparative evidence rather than a quantitative stability ranking.

## 4. Discussion

### 4.1. Influence of Morphology and Anchoring Orientation on Apparent Conjugation Output

The Qubit-based results indicate that nanoparticle morphology influenced the measured apparent conjugation output under the present experimental workflow. Under the tested stock-dispersion conditions, AuNP samples yielded higher apparent nanoparticle-associated DNA output than AuNF samples for both terminal thiol constructs. A plausible explanation is that smoother spherical AuNPs provide a more spatially uniform interfacial environment for thiolated DNA association, whereas branched AuNFs contain heterogeneous local curvature, protruding tip regions, and recessed surface domains that may reduce the uniformity of ligand accessibility and packing [9,11,21,34,35]. In such systems, differences in local curvature may affect not only how densely thiolated strands can approach the surface, but also how the interfacial DNA layer reorganizes during stepwise salt-aging [34,35].

At the same time, these morphology-related effects should not be overstated as the sole explanation for the observed differences. Because the AuNP and AuNF dispersions were used as supplied and were not normalized to identical particle number or total accessible surface area, the present comparison is more appropriately interpreted as a difference in apparent conjugation-related output under matched preparation conditions, rather than a strict particle-normalized comparison of intrinsic loading capacity [11,21].

Differences between the 3′-SH and 5′-SH constructs were also observed within each particle type, with the 5′-SH construct showing slightly higher apparent output than the 3′-SH construct under the present workflow. This result suggests that terminal thiol placement may influence nanoparticle association behavior through sequence-specific effects on terminal accessibility, steric presentation, and local conformational arrangement during surface immobilization [21,25]. Because only one aptamer sequence was evaluated here, this observation should be interpreted as a construct-dependent materials effect rather than a universal rule for all aptamer systems.

### 4.2. Hydrodynamic Size and Zeta Potential as Comparative Interfacial Indicators

The increase in hydrodynamic diameter after aptamer modification is consistent with the formation of a hydrated DNA-associated interfacial layer around the nanoparticles. For both AuNPs and AuNFs, DLS-measured size increased after modification, supporting the view that surface-associated nucleic acid altered the effective hydrodynamic envelope of the particles. However, these DLS differences should be interpreted cautiously, because hydrodynamic diameter reflects not only surface-bound DNA, but also solvation effects, interparticle interactions, and the sensitivity of the technique to diffuse interfacial layers [23,24].

Zeta potential measurements showed a clear shift toward more negative values after aptamer modification, which is likewise consistent with an increased contribution from the negatively charged phosphate backbone of nanoparticle-associated DNA [23,24]. Notably, despite differences in apparent Qubit-based DNA output, the final zeta potential values of the modified AuNP and AuNF systems converged to a relatively narrow range near −40 mV. One plausible interpretation is that, once a sufficiently continuous DNA-associated interfacial layer is established, the measured outer electrokinetic environment becomes increasingly dominated by the exposed DNA phosphate backbone rather than by the original citrate-capped gold surface alone [23,24].

At the same time, this convergence should not be overinterpreted as definitive evidence that all samples reached identical surface saturation or identical ligand coverage. Zeta potential reflects the electrokinetic behavior of the outer interfacial layer and is influenced by hydration, ionic environment, and charge distribution, rather than providing a direct quantitative readout of the total number of associated ligands [23,24]. Therefore, in the present study, zeta potential is most appropriately regarded as a supplementary comparative indicator of interfacial modification status rather than a direct proxy for absolute loading density.

### 4.3. Apparent Qubit-Based DNA Association Output Across Particle Types

The Qubit-based mass-balance results showed that the apparent amount of nanoparticle-associated DNA differed across particle type and terminal thiol configuration. In absolute mass terms under the present workflow, AuNP–5′SH showed the highest apparent output, followed by AuNP–3′SH, AuNF–5′SH, and AuNF–3′SH. From a materials-preparation perspective, these differences are relevant because the total amount of DNA remaining associated with the particles after standardized purification can influence subsequent colloidal response and interface-dependent analytical behavior [11,21,33].

However, the present values should not be interpreted as direct measurements of intrinsic maximal surface loading capacity. First, the AuNP and AuNF stock dispersions were not normalized to the same particle number or total accessible surface area. Second, the Qubit-based calculation reflects the amount of DNA remaining nanoparticle-associated after repeated centrifugation and washing, rather than a direct distinction between irreversibly chemisorbed DNA and all weakly associated DNA species. For this reason, the data are best understood as comparative apparent outputs of the tested stock systems under a standardized purification workflow [11,21].

Within this scope, the Qubit-based results are still informative because they provide a practical and internally consistent comparative readout across all four groups. Nevertheless, more rigorous particle-normalized or surface-area-normalized loading analysis, together with direct spectroscopic confirmation of surface binding, will be required in future work to define intrinsic loading behavior more precisely.

### 4.4. Qualitative Visual Response Under Electrolyte Challenge

The salt-challenge experiment showed that aptamer-modified AuNPs and AuNFs exhibited improved qualitative visual tolerance to elevated ionic strength compared with their bare counterparts. Bare AuNPs and AuNFs showed obvious color change and visible aggregation-related destabilization after NaCl addition, whereas the aptamer-modified systems did not display comparable macroscopic change under the same conditions. This result is consistent with the expected stabilizing contribution of a surface-associated DNA layer, which can provide both electrostatic and steric resistance against salt-induced aggregation in citrate-based gold colloids [15,16,17,25].

The behavior of the AuNF systems is particularly noteworthy because branched or anisotropic nanostructures often possess more heterogeneous surface curvature and interfacial environments than smoother spherical particles [4,5,6,27,34,35]. Such heterogeneity may increase the sensitivity of the bare AuNF dispersions to ionic perturbation, while DNA association can partially buffer this response by modifying the effective interfacial charge and steric environment [34,35]. At the same time, the present experiment was based on photographic observation rather than time-resolved DLS or UV–Vis monitoring. Therefore, these data should be interpreted as qualitative comparative evidence of improved salt tolerance, rather than as a quantitative ranking of colloidal stability.

Within the scope of this study, the electrolyte-challenge experiment is best understood as a simple visual screen of whether aptamer association improved macroscopic colloidal robustness under the tested conditions. More rigorous kinetic characterization will be required in future work to resolve how morphology, terminal thiol placement, and interfacial DNA organization quantitatively influence stability over time.

## 5. Conclusions

In this study, thiolated DNA aptamer constructs bearing 3′-SH or 5′-SH terminal modification were conjugated onto commercially supplied gold nanospheres (AuNPs) and gold nanoflowers (AuNFs) using a matched stepwise salt-aging workflow. Under the tested stock-dispersion conditions, the AuNP systems showed higher apparent Qubit-based DNA association output than the AuNF systems, while modest differences were also observed between the 3′-SH and 5′-SH constructs. Aptamer modification was further associated with increased hydrodynamic diameter, a shift of zeta potential toward more negative values, retention of particle morphology, and improved qualitative visual tolerance to salt-induced aggregation relative to bare particles.

These results should be interpreted within the scope of a preliminary materials-level comparative evaluation rather than a complete biosensor validation study. Because the commercially supplied AuNP and AuNF dispersions were not normalized to identical particle number or total accessible surface area, the reported DNA-associated mass and apparent conjugation yield represent comparative outputs under standardized preparation conditions rather than intrinsic loading capacities. In addition, the present evidence for aptamer conjugation is based on Qubit-assisted mass balance together with DLS, zeta potential, TEM, and visual salt-challenge behavior, and therefore remains indirect rather than direct spectroscopic confirmation of Au–S bonding.

Within these boundaries, the present work provides a convenient preliminary framework for comparing thiolated aptamer conjugation behavior on gold nanostructures and offers an initial materials-level basis for future optimization of glyphosate aptamer-functionalized sensing systems. Future work should incorporate particle-normalized loading analysis, direct surface-chemistry characterization, quantitative stability measurements, and functional target-binding or sensing validation to define structure–interface–performance relationships more rigorously.

## Figures and Tables

**Table 1:** Major instruments employed in the experiments.

Instrument	Model	Manufacturer
Analytical balance	OHAUS-CP114	OHAUS Instruments (Shanghai) Co., Ltd., Shanghai, China
Transmission electron microscope (TEM)	JEM-2100	JEOL Ltd., Akishima, Tokyo, Japan
Dynamic light scattering/Zeta potential	Nano ZS	Malvern Panalytical Ltd., Malvern, Worcestershire, UK
Fluorometer	Qubit 4.0	Thermo Fisher Scientific Inc., Waltham, MA, USA
Refrigerated centrifuge	5430R	Hunan Xiangyi Laboratory Instrument Development Co., Ltd., Changsha, Hunan, China

**Table 2:** Qubit-based apparent DNA association output and apparent conjugation yield for AuNPs and AuNFs under matched preparation conditions.

Sample	DNA Input Concentration, $C_{in}$ (ng/µL)	DNA Input Volume, $V_{in}$ (µL)	Total DNA Input (ng)	Supernatant ssDNA Concentration, $C_{sup}$ (ng/µL)	Supernatant Volume, $V_{sup}$ (µL)	Supernatant ssDNA (ng)	Conjugated DNA (ng)	Conjugation Yield (%)
AuNP–3′SH	288.9	10	2889.0	0.47 ± 0.04	1190	559.3 ± 47.4	2329.7 ± 47.4	80.65 ± 1.64
AuNP–5′SH	288.9	10	2889.0	0.37 ± 0.05	1190	440.3 ± 57.1	2448.7 ± 57.1	84.76 ± 1.98
AuNF–3′SH	288.9	10	2889.0	0.81 ± 0.08	1190	963.9 ± 97.0	1925.1 ± 97.0	66.64 ± 3.36
AuNF–5′SH	288.9	10	2889.0	0.64 ± 0.03	1190	761.6 ± 39.3	2127.4 ± 39.3	73.65 ± 1.36

Values are reported as mean ± SD from four independent measurements (*n* = 4).

**Table 3:** Hydrodynamic diameter and zeta potential of AuNPs and AuNFs before and after aptamer conjugation.

Type	Hydrodynamic Diameter (nm)	Zeta Potential (mV)
AuNPs	70.41 ± 1.10	−28.40 ± 0.64
AuNPs-3′SH	118.30 ± 2.04	−40.80 ± 0.59
AuNPs-5′SH	119.10 ± 1.06	−41.50 ± 0.66
AuNFs	70.35 ± 1.27	−36.00 ± 0.45
AuNFs-3′SH	108.80 ± 2.67	−40.55 ± 0.66
AuNFs-5′SH	115.30 ± 3.18	−39.98 ± 0.89

Values are reported as mean ± SD (*n* = 4), with units of nanometers (nm) for hydrodynamic diameter and millivolts (mV) for zeta potential.

## Data Availability

All data supporting the findings of this study are available within the article.
